# The Prospective Synergy of Antitubercular Drugs With NAD Biosynthesis Inhibitors

**DOI:** 10.3389/fmicb.2020.634640

**Published:** 2021-01-26

**Authors:** Kyle H. Rohde, Leonardo Sorci

**Affiliations:** ^1^Burnett School of Biomedical Sciences, College of Medicine, University of Central Florida, Orlando, FL, United States; ^2^Division of Bioinformatics and Biochemistry, Department of Materials, Environmental Sciences and Urban Planning, Polytechnic University of Marche, Ancona, Italy

**Keywords:** isoniazid, pyrazinamide, nicotinamide, ethionamide, delamanid, tuberculosis, toxin-antitoxin system, NAD biosynthesis inhibition

## Abstract

Given the upsurge of drug-resistant tuberculosis worldwide, there is much focus on developing novel drug combinations allowing shorter treatment duration and a lower toxicity profile. Nicotinamide adenine dinucleotide (NAD) biosynthesis targeting is acknowledged as a promising strategy to combat drug-susceptible, drug-resistant, and latent tuberculosis (TB) infections. In this review, we describe the potential synergy of NAD biosynthesis inhibitors with several TB-drugs in prospective novel combination therapy. Despite not directly targeting the essential NAD cofactor’s biosynthesis, several TB prodrugs either require a NAD biosynthesis enzyme to be activated or form a toxic chemical adduct with NAD(H) itself. For example, pyrazinamide requires the action of nicotinamidase (PncA), often referred to as pyrazinamidase, to be converted into its active form. PncA is an essential player in NAD salvage and recycling. Since most pyrazinamide-resistant strains are PncA-defective, a combination with downstream NAD-blocking molecules may enhance pyrazinamide activity and possibly overcome the resistance mechanism. Isoniazid, ethionamide, and delamanid form NAD adducts in their active form, partly perturbing the redox cofactor metabolism. Indeed, NAD depletion has been observed in *Mycobacterium tuberculosis* (Mtb) during isoniazid treatment, and activation of the intracellular NAD phosphorylase MbcT toxin potentiates its effect. Due to the NAD cofactor’s crucial role in cellular energy production, additional synergistic correlations of NAD biosynthesis blockade can be envisioned with bedaquiline and other drugs targeting energy-metabolism in mycobacteria. In conclusion, future strategies targeting NAD metabolism in Mtb should consider its potential synergy with current and other forthcoming TB-drugs.

## Introduction

Tuberculosis (TB), caused by *Mycobacterium tuberculosis* (Mtb), is the leading infectious cause of mortality, with an estimated 1.2 million deaths and 10 million new cases in 2019, and about a quarter of the world’s population latently infected (WHO, 2020). The shortcomings of currently available TB drugs are a major factor underlying the ongoing TB crisis. The current “short course” regimen involves a cocktail of multiple front-line drugs administered for 6 months. In a sobering finding, Malherbe et al. (2016) reported detection of *Mtb* RNA within nonresolving granulomas in patients who had been declared cured after standard 6-month treatment using PET-CT imaging. This highlights the inability of current drug regimens to effectively eradicate phenotypically tolerant subpopulations of *Mtb* persisters sequestered within granulomas, the hallmark lesions of TB disease. Further, exacerbating the challenge of treating TB is the COVID-19 pandemic which is hampering the TB detection and the increasing emergence of drug resistance. According to the latest report on TB epidemic, in 2019, nearly 400,000 people developed multidrug-resistant TB (MDR-TB), which is caused by strains resistant to the two first-line drugs rifampicin (RIF) and isoniazid (INH) with a treatment success rate of 57% globally (WHO, 2020). Even more concerning are extensively drug resistant TB (XDR-TB), resistant to RIF, INH, fluoroquinolones, and at least one of the three classes of injectables (amikacin, kanamycin, and capreomycin), and totally drug resistant TB (TDR-TB), which are not susceptible to known drugs (Udwadia et al., 2012). Thus, there is an urgent need for new multi-drug regimens with potent bactericidal activity against dormant persisters and drug-resistant mycobacteria. Identifying novel antimicrobials with unique mechanisms of action that can synergize with drugs currently in use or at late stages of development is a promising strategy to address this need. Prompted by our efforts to develop inhibitors of nicotinamide adenine dinucleotide (NAD) biosynthesis, this review seeks to highlight scientific evidence that chemical perturbation of this pathway could be an effective component of new TB drug regimens.

### Isoniazid and NAD Metabolism

**Figure 1:**
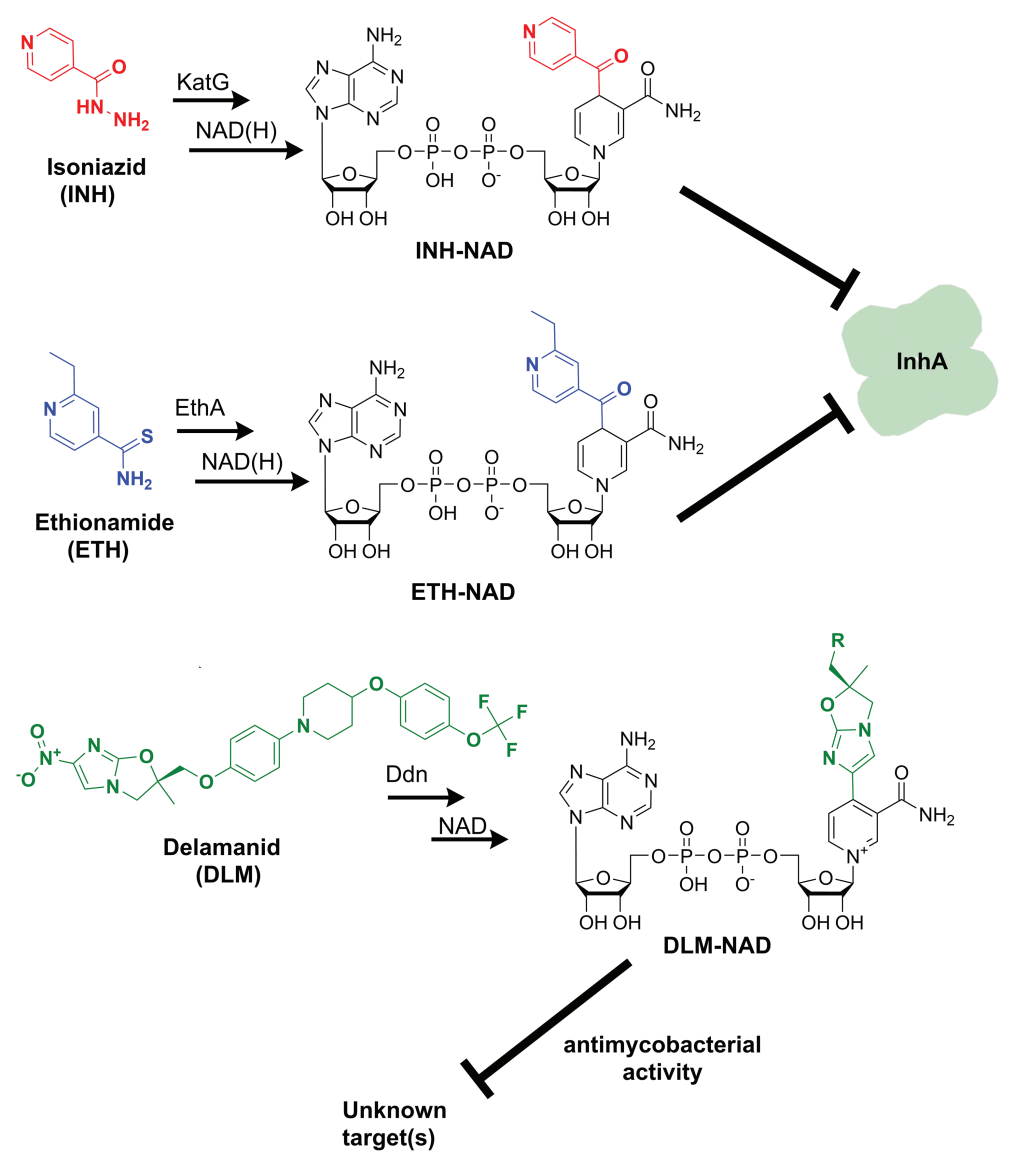
Formation of nicotinamide adenine dinucleotide (NAD) chemical adducts by tuberculosis (TB) drugs. Prothionamide, a close chemical analog of ethionamide, undergoes the same transformation. Pretomanid, belonging to the same class of nitroimidazoles as delamanid, may combine with NAD as well. Their structures have been omitted for clarity.

Discovered back in 1952, isoniazid, also known as isonicotinic acid hydrazide (INH), remains today one of the major first-line antituberculosis drugs. INH only kills actively replicating bacteria, with a minimum inhibitory concentration (MIC) against the slowly growing *M. tuberculosis* of 0.05 μg ml^−1^. In contrast to the exquisite activity of INH on Mtb, most mycobacteria are only susceptible to INH concentrations over 1 μg/ml (Zhang and Young, 1993). The precise target and mechanism of action of INH have eluded the grasp of science for decades. A combination of biochemical, genetic, and X-ray crystallography studies finally concluded that INH is a prodrug involved in inhibiting the biosynthesis of mycolic acid, an essential cell wall component of Mtb (Winder et al., 1970). This prodrug’s most accepted mechanism of action requires its activation into an acyl radical by the catalase-peroxidase KatG enzyme (Lei et al., 2000). The radical reacts with NAD to form an INH-NAD adduct (Figure 1), which, in turn, inhibits the FASII enoyl-ACP reductase InhA, leading to mycobacterial cell death (Rawat et al., 2003). The elucidation of the INH action mechanism has been controversial, and alternative target pathways, including DNA and lipid biosynthesis (Russe and Barclay, 1955; Ebina et al., 1961; Gangadharam et al., 1963), cell division (Barclay et al., 1953), or altered NAD metabolism (Bekierkunst, 1966), have been proposed in early reports.

The emergence of NAD enzyme cofactor biosynthesis as a promising antimycobacterial target pathway warrants a reevaluation of the metabolic interplay between INH and NAD. The perturbation of NAD metabolism by isoniazid was an early finding related to isoniazid action and resistance. In 1966, Bekierkunst reported that in *M. tuberculosis* H37Rv, a decrease in NAD content could be observed after only 4 h of exposure to isoniazid, rising to 50% after 6 h (Bekierkunst, 1966). Ten years later, Jackett et al. (1977) established that the NAD content declined with increasing drug concentration and increased exposure. These results, though, did not support a direct causal link between NAD depletion and INH’s antibacterial activity, mainly because the NAD drop was marginal at the low bactericidal concentration of INH.

Despite the fact that NAD pool perturbation appears to be an incontrovertible secondary effect of INH action, this link has not been thoroughly elucidated. Several conjectures have been advanced for explaining the mechanism of NAD drop in the presence of INH. These hypotheses have been widely questioned or disproved. For example, it was suggested that NAD loss was as a result of leaks in the cell wall and membrane due to the inhibition of mycolate synthesis, i.e., a post-antibiotic effect (Winder et al., 1971); however, NAD level restored in cells exposed to INH for 24 h, despite the continued inhibition of growth (Jackett et al., 1977). It was also proposed that NAD depletion by INH could be the indirect effect of a NAD glycohydrolase activation (Bekierkunst and Bricker, 1967). Yet, this hypothesis did not explain resistance to isoniazid, as the effects on NAD were equivalent in isoniazid susceptible and resistant strains of *M. tuberculosis* (Sriprakash and Ramakrishnan, 1970).

More clues into the relationship between NAD and INH had to wait for in depth studies of INH resistance. The primary mechanism of resistance to INH results from mutations that either depress its activator (KatG) activity or overexpress/alter InhA, the target of INH-NAD adduct (Vilcheze and Jacobs, 2007). KatG mutants exhibit decreased or complete loss of catalase and peroxidase activity. Because these enzymatic activities are essential for *M. tuberculosis* defense against reactive oxygen species (ROS) and virulence *in vivo* (Pym et al., 2002; Ng et al., 2004), polymorphism in *katG* may result in a loss of fitness to the bacterium. On the other hand, among a set of overexpressed genes during NAD starvation, KatG came up with the highest upregulation (nearly 7-fold; Vilcheze et al., 2010), pointing to NAD depletion as a trigger to ROS production. Hence, INH and prospective NAD inhibitors may potentiate their effects and reduce the emergence of KatG-mediated resistance mechanisms. In support of this assumption, antagonism was reported between isoniazid and nicotinamide (Jordahl et al., 1961), the primary precursor of mycobacterial NAD salvage pathway.

An alternative mechanism of INH resistance in Mtb is based on defects in the *ndh* gene (Rv1854c; Lee et al., 2001), which encodes a type II NADH dehydrogenase. This oxidase transfers electrons from NADH to the respiratory chain without proton translocation. The *ndh*-mediated mechanism of resistance was first described in *Mycobacterium smegmatis* and *M. bovis* (Miesel et al., 1998; Vilcheze et al., 2005). As expected, the activity of NdhII mutants was compromised, yielding an increased NADH cellular content and NADH/NAD ratio than wild-type (Vilcheze et al., 2005). In the same study, the authors demonstrated that the accumulation of NADH, the native substrate of InhA, acted as a competitive inhibitor for binding of the INH-NAD adduct to InhA. Despite an impaired NADH to NAD oxidation, the increased NADH/NAD ratio was only achieved by NADH elevation, while NAD remained relatively constant. As a result, the total amount of NAD(H) pyridine nucleotides in *ndh* mutants grew by 10–50% (Vilcheze et al., 2005). This implies that (i) NAD cofactor homeostasis is tightly regulated in tubercle bacilli and (ii) an increased NAD biosynthesis is necessary to avoid a dramatic NAD drop in *ndh* mutants.

**Figure 2:**
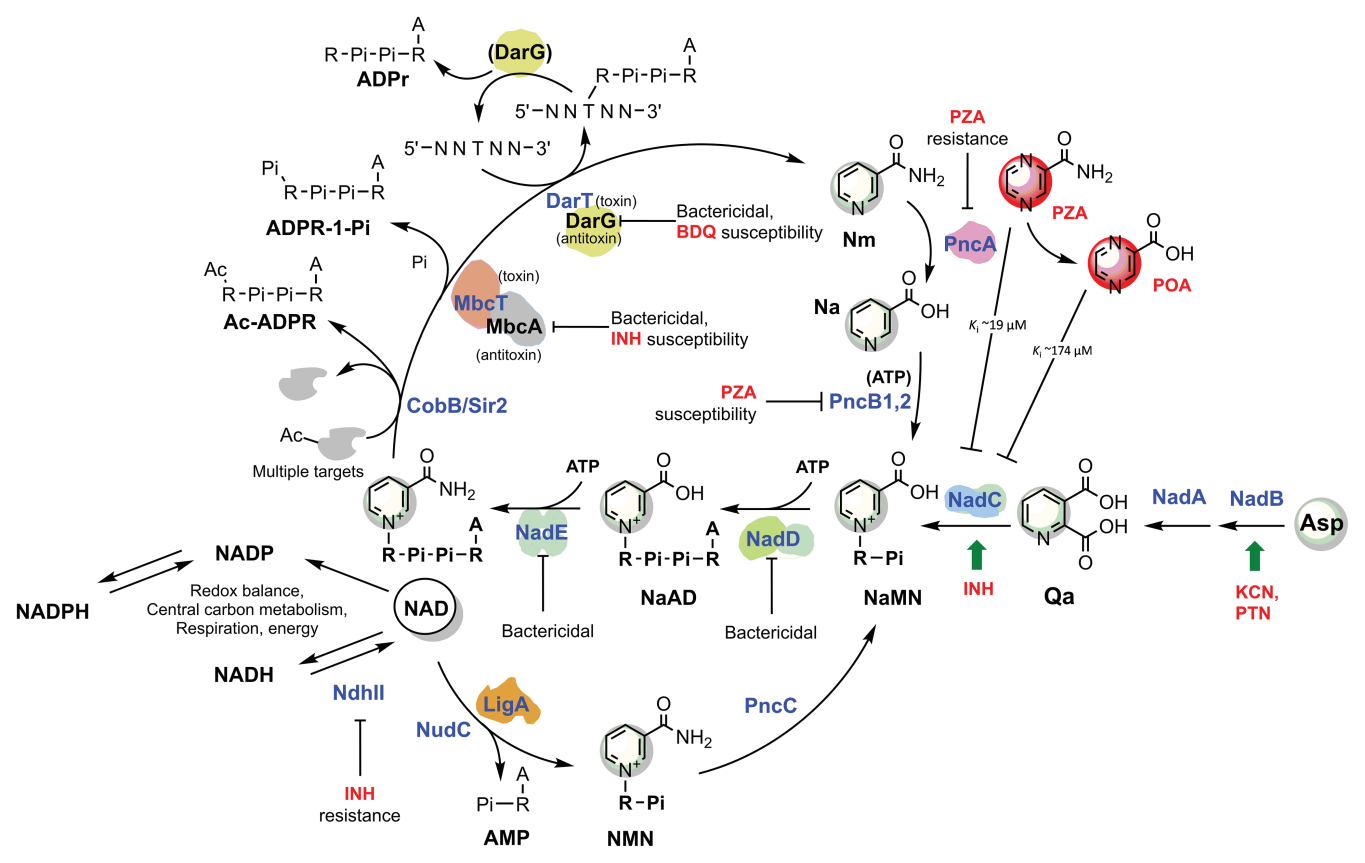
The metabolic interplay between TB drugs and NAD pathways in *Mycobacterium tuberculosis*. Enzymes (blue) are identified with their gene name, metabolites’ names are in black, and TB drugs or inhibitors are in red. The effects of targeted enzymes’ inhibition/downregulation on bacterial growth or drug efficacy are shown. Green arrows indicate upregulated enzymes upon drug exposure, according to transcriptional analyses. Proteins with available 3D structures are sketched based on their biological quaternary assembly. Enzyme abbreviations: NadB (Rv1595): ASPOX, L-aspartate oxidase; NadA (Rv1594): QSYN, quinolinate synthetase; NadC (Rv1596): QaPRT, quinolinate phosphorybosyltransferase; PncB1 (Rv1330c): NaPRT1, nicotinate phosphoribosyltransferase; PncB2 (Rv0573c): NaPRT1, nicotinate phosphoribosyltransferase; NadD (Rv2421c): NaMNAT, NaMN adenylyltransferase; NadE (Rv2438c): NADS, glutamine-dependent NAD syntetase; PncA (Rv2043c): NMASE, nicotinamide deamidase; PncC (Rv1901): NMND, NMN deamidase; LigA (Rv3014c): NAD-dependent DNA ligase; NudC (Rv3199c): NAD(H) pyrophosphatase; NdhII (Rv1854c); type II NADH:menaquinone oxidoreductase; Sir2/CobB (Rv1151c): NAD-dependent deacetyltransferase. Metabolite abbreviations: NaAD, nicotinic acid adenine dinucleotide; Nam, nicotinamide; Na, nicotinic acid; Qa, quinolinic acid; NaMN, nicotinic acid mononucleotide; NMN, nicotinamide mononucleotide; PRPP, phosphoribosyl diphosphate; PZA, pyrazinamide; POA, pyrazinoic acid; BDQ, bedaquiline; INH, isoniazid; PTN, pretomanid; KCN, potassium cyanide.

Consistently, a gene expression analysis of *M. tuberculosis* exposed to INH showed that *nadC* (Rv1596) and *pntAb* (Rv0156) genes are significantly upregulated (Waddell et al., 2004). The gene *nadC* codes for quinolinate phosphoribosyltransferase (QAPRT), the final enzyme in the *de novo* branch of NAD biosynthesis (Waddell et al., 2004; Figure 2), while *pntAB* specifies a component (subunit alpha) of a proton-translocating NAD(P) transhydrogenase transferring reducing equivalents from NADPH to NAD. Supposedly, to counteract INH’s bactericidal action, the two enzymes implement a metabolic response whereby the transhydrogenase elevates the NADH content while NadC replenishes the oxidized NAD cofactor. In this perspective, the blocking of NAD biosynthesis with specific inhibitors could represent not only a novel antimycobacterial strategy but an opportunity to synergize with isoniazid, making the drug more lethal and overcoming some forms of resistance as in *ndh* mutants.

It could be argued that NAD depletion would also impair INH activity as less NAD would be available to form the INH-NAD adduct, thus counteracting this effect. Reasonably, this is not the case as INH-NAD is a tight-binding subnanomolar inhibitor of InhA (*K*_i_ = 0.75 nM; Rawat et al., 2003). We have demonstrated that a drop of NAD(H) content of only 3-fold becomes bactericidal for Mtb (Rodionova et al., 2014). Thus, given that typical bacterial NAD(H) pool concentration is around 1 mM, the residual NAD would be large enough to drive the INH-NAD adduct formation.

Recently, Freire et al. (2019) demonstrated that the toxin component MbcT of the Toxin-antitoxin MbcTA system is a novel phosphorylase that degrades NAD (Figure 2) and, in the absence of the McbA antidote, reduces mycobacterial survival in macrophages while extending the survival of infected mice. Remarkably, the study revealed a synergistic effect of toxin activity with isoniazid drug in a mouse model of Mtb infection, providing an additional proof-of-concept for INH’s foreseen synergy with NAD synthesis blockers (Figure 2).

### Ethionamide/Prothionamide and Delamanid/Pretomanid: Other NAD Adduct-Forming Prodrugs

Ethionamide (ETH) and its close analog prothionamide (PRO) are interchangeable components of second-line drug regimens used for the treatment of drug-resistant TB. Like its structural analog isoniazid, ETH is a prodrug targeting InhA. However, ETH is activated by a different enzyme, the flavin-dependent monooxygenase EthA (Vannelli et al., 2002). The activated ETH reacts with NAD to yield an ETH-NAD adduct (Figure 1), which subsequently inhibits InhA (Wang et al., 2007). Due to the commonalities in the mechanisms of action and cross-resistance of ETH or PRO and isoniazid, similar conclusions regarding the expected interference of ETH or PRO with NAD metabolism can be drawn.

The multi-drug-resistant TB drug delamanid (DLM) is a recently approved drug that also blocks the synthesis of mycolic acids, thereby destabilizing its cell wall (Matsumoto et al., 2006). DLM undergoes activation by the deazaflavin (F_420_)-dependent nitroreductase (*ddn*) to yield a reactive intermediate. This activated metabolite is considered to play a crucial role in the drug’s bactericidal effect but whether it represents the final toxic derivative and the exact identification of its molecular target are still unknown (Liu et al., 2018). Strikingly, it has been recently established that after activation, DLM can combine with NAD to form a DLM-NAD adduct (Figure 1), which plays an essential role in the antimycobacterial action of DLM (Hayashi et al., 2020). Hence, delamanid is another TB drug that interferes with the pathogen’s NAD homeostasis and that can potentially synergize with the NAD biosynthesis blockade. Similar conjectures can be drawn for the drug pretomanid (PTN), belonging to the same class of nitroimidazoles, but no experimental evidence that pretomanid can combine with NAD is currently available in the literature.

### Pyrazinamide and NAD Metabolism

Pyrazinamide (PZA) drug partners with isoniazid, rifampicin, and ethambutol in the present-day “short-course” TB drug regimen (Mitchison, 1985). Since the discovery of the *in vivo* sterilizing activity in the animal models and humans (Solotorovsky et al., 1952; Yeager et al., 1952), PZA action mechanism largely remained an enigma. Several modes of action have been proposed in the following decades, though mounting evidence opposing such models was promptly put forward (for in-depth reviews, see Anthony et al., 2018; Lamont et al., 2020). These studies identified a diverse range of potential targets, including fatty acid synthesis (Zimhony et al., 2000), membrane energetics and integrity (Wade and Zhang, 2006), protein translation (Shi et al., 2011), and pantothenate biosynthesis (Zhang et al., 2013a; Dillon et al., 2014). More recently, aspartate decarboxylase PanD, required for Coenzyme A (CoA) biosynthesis, emerged as a convincing target of pyrazinoic acid (PAO), the bioactive form of PZA (Gopal et al., 2019). Remarkably, instead of inhibiting its target protein’s function, as most antibacterials do, PAO functions as a target degrader (Gopal et al., 2020).

Hereafter, we will focus on the link between pyrazinamide and NAD metabolism, with the intent of uncovering foreseeable synergies between PZA and NAD pathway inhibitors. PZA is a prodrug hydrolyzed to the bioactive POA in the mycobacterial cytoplasm by the *M. tuberculosis* pyrazinamidase/nicotinamidase (PZAse), encoded by *pncA* (Rv2043c; Scorpio and Zhang, 1996). This amidase hydrolyzes nicotinamide vitamin into nicotinate, subsequently converted by PncB1-2 to nicotinate mononucleotide (NaMN), a shared product with NadABC-mediated *de novo* biosynthesis (Figure 2). Due to the redundancy of these pathways, PncA is nonessential for survival and virulence of *M. tuberculosis* (Boshoff et al., 2008; Vilcheze et al., 2010), and loss-of-function mutations represent the predominant mechanism for PZA resistance in clinical isolates (with up to 99.9% frequency; Zhang and Mitchison, 2003). The virulence and fitness of PZA-resistant strains with *pncA* mutations do not seem to be altered (Cheng et al., 2000). However, PncA resistant mutants may be more vulnerable to antibacterials targeting NAD metabolism, and such molecules could help overcome pncA-mediated resistance. Indeed, pncA-mutants would not efficiently salvage the Nm precursor, which is particularly abundant in the human host, nor recycle it out of NAD-consuming reactions (Figure 2). Mycobacteria encode Nm-releasing enzymes using NAD as a substrate that play vital roles in the dynamic regulation of metabolic functions (Figure 2). For example, in *M. tuberculosis*, the NAD-dependent Sir2-like family protein CobB (Rv1151c) influences the DNA architecture by deacetylating nucleoid-associated protein HU, a protein essential for growth (Anand et al., 2017; Green et al., 2018). Similarly to other Sir2-family proteins, Rv1151c is inhibited by Nm (Gu et al., 2009), which can accumulate in PZAse-defective Mtb strains. As also discussed in the next paragraph, the Mtb DarTG toxin-antitoxin system consumes NAD to mediate the reversible DNA ADP-ribosylation, a process whose biological significance is still poorly understood (Zaveri et al., 2020). If activated intracellularly, two other toxins, like the abovementioned MbcT and the TNT necrotizing toxin (Sun et al., 2015), may challenge intracellular Mtb NAD homeostasis. An uncharacterized NAD glycohydrolase activity, along with its heat-labile inhibitor, have been identified in Mtb crude extracts in the 1960s (Gopinathan et al., 1964, 1966), which likely correspond to the TNT toxin and its inhibitor IFT (Sun et al., 2015).

Thus, even though Nm salvage or recycling does not significantly affect tubercle bacillus survival or pathogenicity, its absence may impair the bacterial capacity to maintain NAD homeostasis under specific stress conditions that yield a sudden drop in NAD content. Such stress conditions may also be induced by PZA drug itself (Figure 2), which has been reported being an inhibitor of *de novo* NAD biosynthesis enzyme quinolinate phosphoribosyltransferase (QAPRT; Sharma et al., 1998; Kim et al., 2014). In keeping with this proposition, mutations in NAD pathways and energy production cause increased PZA susceptibility. Of note, mutants in *pncB1*, involved in NAD synthesis, are 5-fold more susceptible to PZA (Zhang et al., 2013b). Other mutants exhibiting higher PZA susceptibility were defective in energy production and include mutations in NADH dehydrogenase subunits H and N (*nuoH* and *nuoN*), nitrate reductase *narH*, and formate dehydrogenase *fdhF* (Zhang et al., 2013b). Given these premises, it seems worthwhile to test whether antimycobacterials that target NAD biosynthesis potentiate the effect of PZA and improve the treatment of TB.

### The Metabolic Interplay Between NAD and ATP Metabolism: Bright Prospects for Respiratory and NAD Synthesis Inhibitors

It has been assessed that nearly 17% of the central metabolism enzymatic reactions that are essential for the survival of *M. tuberculosis* require the NAD(H) cofactor and its phosphorylated derivative NADP(H) (Beste et al., 2007). Among these are the key pathways required to produce ATP as NAD(H) is the primary entry electron donor in the respiratory chain and the oxidant driving the glycolysis. Thus, a NAD(H) pool decrease induces a glycolytic slowdown and rapid shutdown of the electron transfer chain, ultimately compromising ATP synthesis from both sources.

In turn, NAD(P) biosynthesis is a highly ATP-dependent pathway (Figure 2). Each of the three universal enzymatic steps converting NaMN precursor to NADP requires an ATP molecule (Sorci et al., 2010b). Moreover, PncB-driven NaMN synthesis from Na and PRPP is strongly activated by the PncB ATPase activity (Vinitsky and Grubmeyer, 1993), and additional ATP molecules are required for recycling the ADP-ribose moiety into the PRPP precursor. The biogenesis and homeostasis of the NAD pool and ATP are undoubtedly interlinked aspects of energy metabolism, and their simultaneous targeting in innovative drug combinations may hold the promise for potential synergy and overcome resistance mechanisms. In line with this concept, Vilcheze et al. (2010) showed that both *M. tuberculosis* and *M. bovis de novo* NAD synthesis mutants starved for nicotinamide, i.e., in a metabolic status mimicking NAD synthesis inhibition, strongly upregulate ATP synthetase subunits *b*, *d*, and *c*, the latter being targeted by the recently approved bedaquiline drug (Andries et al., 2005). This implies that electron transport is impaired by limiting NAD and the ATP synthetase upregulation represents an attempt to restore electron transport and oxidative phosphorylation efficiency. Consistently, the transcriptional profiles from *M. tuberculosis* during exposure with NadE inhibitors resembled those induced by respiratory inhibitors (Boshoff et al., 2008). Conversely, respiratory poisons such as potassium cyanide and pretomanid positively affected the transcription of Mtb *nadB* (Manjunatha et al., 2009), the first and rate-limiting gene of *de novo* NAD biosynthesis, regulated by classic feedback inhibition of NAD (Seifert et al., 1990; Tedeschi et al., 1999).

Although they require additional supporting evidence, these observations based on transcriptional profiling emphasize the strict interdependence between ATP and NAD biogenesis and homeostasis in Mtb. Thus, we propose that innovative antimycobacterial strategies disrupting energy metabolism should target both of these two pathways for the best outcome. The latest developments on inhibitors interfering with energy-metabolism in *M. tuberculosis* are covered in comprehensive reviews (Thompson and Denny, 2019; Appetecchia et al., 2020). Not surprisingly, partial depletion of Mtb DarG (Rv0060), the hydrolase antitoxin reversing DarT-catalyzed DNA ADP-ribosylation (Jankevicius et al., 2016), sensitizes the mycobacterial cell to drugs targeting respiration (i.e., bedaquiline) and DNA metabolism (Zaveri et al., 2020). DarT (Rv0059) consumes NAD as an ADP-ribose donor and interacts with other proteins involved in DNA replication and repair, whereby additional NAD is drained by the activity of the NAD-dependent DNA ligase LigA (Srivastava et al., 2005). Although the Mtb DarTG toxin-antitoxin system’s exact function remains unsolved, the essentiality of DarG and its direct involvement with DNA-repair proteins argues in favor of a significant role in preserving the genome integrity under stress conditions (Ramage et al., 2009). As an obligate human intracellular pathogen, *M. tuberculosis* has evolved complex defenses to endure the stresses experienced during persistent infection. As exemplified by the DarTG Toxin-Antitoxin system, NAD consumption is required to fuel the Mtb resilience under stress conditions, thus reaffirming bacterial NAD replenishment as an attractive target pathway.

## Concluding Remarks and Perspectives

In 1945, nicotinamide was serendipitously discovered to have antituberculosis properties (Chlorine, 1945). This prompted the testing of other pyridine derivatives for their antimycobacterial effects and paved the way for discovering isoniazid and pyrazinamide front-line drugs. Since these agents were much more effective than nicotinamide, further efforts were focused on uncovering their mechanism of action, obscuring the interest in nicotinamide. However, the elucidation of how the vitamin nicotinamide exerts its direct antituberculosis action, which must differ from its renowned derivatives, is of paramount importance as it may disclose novel vulnerable targets tied to the mycobacterial NAD metabolism. For example, Mtb CobB is a NAD-dependent deacetylase member of the SIR2 family of proteins that are notoriously inhibited by nicotinamide (Hu et al., 2014). Of relevance, nicotinamide inhibits *Pf*Sir2 activity and intra-erythrocytic growth of *Plasmodium falciparum* (Prusty et al., 2008). An *in vitro* antileishmanial activity is also reported for nicotinamide, partially mediated by direct inhibition of the *Leishmania* Sir2 homolog (Sereno et al., 2005).

Despite the presence of two PncB isozymes, it is well established that Mtb accumulates Na, and its excess is excreted into the culture media, thus allowing it to be detected using the niacin test. Curiously, this metabolic feature may have evolved to respond to high nicotinamide’s toxicity rather than resulting from an intrinsic inability to process niacin.

The recent discoveries of DarTG, MbcTA, and TNT-IFT toxin-antitoxin systems uncovered additional NAD-consuming enzymes lethal to mycobacteria in the absence of functional antitoxins. Indeed, small inhibitors aiming at disrupting such complexes or inactivating the essential “antidotes” are promising antimycobacterial strategies (Williams and Hergenrother, 2012) that will surely mark future TB research. On the other hand, host NAD is a crucial molecule for TB immunity (Singhal and Cheng, 2018). Recent studies have shown that Mtb is capable of counteracting host defenses by subverting immune cells NAD metabolism. In detail, this could be achieved by modulation of human NAD-consuming proteins such as Sirtuins (Cheng et al., 2017; Bhaskar et al., 2020) or by direct depletion of host NAD levels through the TNT toxin (Pajuelo et al., 2020). The emerging roles of NAD and Sirtuin biology in host immune cells could be exploited to develop adjunct host-directed therapies against TB.

This review outlined the metabolic interplay between multiple TB drugs and the NAD cofactor, a crucial molecule for the Mtb growth during acute infections and persistence in a dormant state (Boshoff et al., 2008; Vilcheze et al., 2010; Kim et al., 2013; Rodionova et al., 2014, 2015). The perturbation of NAD homeostasis by these TB drugs is not, alone, bactericidal. However, it represents collateral damage that could be exploited to synergize with prospective drugs that specifically deplete NAD. Indeed, NAD biosynthesis is an established antiinfective target pathway not only in mycobacteria, but it has also been validated in other bacterial pathogens (Sorci et al., 2009, 2010a, 2013; Huang et al., 2010; Orsomando et al., 2016), the protozoan Plasmodium (O’Hara et al., 2014) and Leishmania (Mandal et al., 2019), and the flatworm Schistosoma parasite (Schultz et al., 2020). In mycobacteria, a detailed analysis of the NAD salvage vs. *de novo*-synthesis pathway concluded that an inhibitor must target either NadD or NadE, which sequentially catalyze the last two shared steps of NAD synthesis (Boshoff et al., 2008). By combining a target-directed and phenotypic screen, our group successfully developed antimycobacterial benzimidazolium compounds targeting *Mt*NadD (Osterman et al., 2019), while other teams optimized biaryl tethered dimers or urea-sulfonamide analogs directed at *Mt*NadE and inhibiting Mtb cell growth in the medium-micromolar range (Boshoff et al., 2008; Wang et al., 2017). A limitation of these studies is that the inhibitors were initially identified for orthologous enzymes from other bacteria (Rodionova et al., 2015; Wang et al., 2017). Thus, computationally-driven, large-scale high-throughput screening on *Mt*NadD and *Mt*NadE may be necessary for identifying more potent mycobacterial NAD synthesis inhibitors that can advance to a drug status.

## Author Contributions

LS designed this study and wrote the manuscript. KR critically reviewed the manuscript and contributed to its final writing. Both the authors contributed to the article and approved the submitted version.

### Conflict of Interest

The authors declare that the research was conducted in the absence of any commercial or financial relationships that could be construed as a potential conflict of interest.
